# Arabic Validation of the Pragmatic Language Skills Inventory to Assess Pragmatic Language Development in Preschoolers with and without Pragmatic Language Impairment

**DOI:** 10.3390/children9060809

**Published:** 2022-05-31

**Authors:** Ahmed Alduais, Fawaz Qasem, Hind Alfadda, Najla Alfadda, Lujain AlAmri

**Affiliations:** 1 Department of Human Sciences (Psychology), University of Verona, Lungadige Porta Vittoria 17, 37129 Verona, Italy; 2 Department of English, University of Bisha, Al-Namas 67714, Saudi Arabia; faqasem@ub.edu.sa; 3 Department of Curriculum and Instruction, King Saud University, Riyadh 11362, Saudi Arabia; 4 Department of English Language and Translation, King Saud University, Riyadh 11451, Saudi Arabia; nalfadda@ksu.edu.sa; 5 Speech Pathology Division, Jeddah Institute for Speech and Hearing and Medical Rehabilitation, Jeddah 23432, Saudi Arabia; lujain@jish.med.sa

**Keywords:** pragmatic language development, pragmatic language impairment, preschoolers, Pragmatic Language Skills Inventory, assessment, diagnosis, validation, Arabic

## Abstract

Objective: An individual’s articulation of pragmatic language development (PLD) signifies successful social interaction with others. Therefore, it is important to detect early pragmatic language impairment (PLI), whether as a primary disorder or as a symptom of other neurodevelopmental disorders. This study reports on validating the Arabic version of the Pragmatic Language Skills Inventory (A-PLSI). Methods: The PLSI was subjected to various validation stages before the A-PLSI was created. To assess PLD in preschoolers with and without psychiatric histories, 264 preschoolers were assessed in several cities in Saudi Arabia by their teachers and speech–language pathologists. Results: The results of this study included three key findings. First, the established psychometric features, including construct validity, criterion-related validity, and (confirmatory) factor analysis, all reported a high level of measurability to consider the A-PLSI a valid instrument for assessing PLD in school settings and diagnosing PLI in clinical settings. Second, the A-PLSI provided empirical evidence by identifying children with and without PLI, documenting their progress on pragmatic language ability, and distinguishing between preschool children in school and clinical settings. In addition, the A-PLSI approved the typical norm that the older the children, the higher their level of PLD: the data showed higher performance for children aged 6–7 compared to the lower PLD level of children aged 4–5. Conclusion: The present study contributes to the existing literature on PLD assessment in a school setting and PLI diagnosis in a clinical setting. More importantly, it adds a new validated tool to the few available instruments in Arabic to assess PLD and diagnose PLI in Arabian contexts.

## 1. Introduction

Pragmatics plays a major role in children’s development of language competencies and social communication [1]. It is a linguistic domain concerned with the appropriate use of language across various social contexts that provides for a listener’s precise and close interpretation of the speaker’s intentions and references [2]. Pragmatics is a field where language rules are applied in social interactions for communication; these language rules are used to express communicative intentions during the conversation [3]. It focuses on how language is used in communication and interaction in a certain context [4]. Furthermore, pragmatics is also a behaviour that covers the emotional and communicative aspects of social interaction [5]. Several studies have examined the pragmatic skills of preschool children, typically exploring the children’s ability to use language for different purposes or communicative intentions (e.g., asking, arguing, suggesting) and conversational skills [6,7,8]. It has also been commonly noticed that children are sensitive to social norms, including the use of language in various contexts [9].

Several studies have addressed the phenomenon of pragmatic development disabilities in preschool and primary school children and the relationship between social interaction and communication problems with pragmatic development problems. In contrast, children who use appropriate pragmatic communication skills usually have successful social interactions with their peers, family members, and teachers [10]. In the same line, another researcher found that students with intellectual disabilities and autism had a lower level of pragmatic language skills. However, students with intellectual disabilities had a higher pragmatic language skill than students with autism [11].

Studies have shown that speech and language development disabilities are linked with challenging behaviours and social skills [12,13]. It has been shown that most children with autism with communication deficits use challenging behaviours to communicate in their school settings (e.g., requesting and rejecting communicative functions) [14]. Several studies have indicated that children who have been neglected or maltreated often demonstrate difficulties with pragmatic skills, such as the use of language in social and communication situations [15,16,17,18,19].

Various assessment tools have emerged from testing the pragmatic development skills and the interactions of preschool children. For instance, the Language Use Inventory (LUI) is a parent-report measure, considering that parents and/or caregivers can perform this assessment since they interact with the child for a long time [6]. Furthermore, the LUI is an inventory in which parents and caregivers assess the child’s language at an early age—18- to 47-months-old—and assessment and intervention can be considered based on the family report [6]. Both the LUI and the Pragmatic Language Skills Inventory (PLIS) [20] are informal assessment tools originating in English for measuring the PLD of preschool children, with the first one focusing on early-age preschoolers. Unlike the LUI, which has been translated to several languages, the PLSI has been translated only to Turkish and adapted and standardised in Turkey, with 1383 students aged between 5 and 12 in grades 1–4. A conventional item analysis of the Turkish Version of the Pragmatic Language Skills Inventory (TV-PLSI) showed that all values are acceptable. The correlation of the TV-PLSI subscale standard scores was between 0.71 and 0.81, while the correlation of TV- PLSI subscales with the Pragmatic Language Skills Index was between 0.76 and 0.84 [21]. Recently, another study used the PLSI to compare the pragmatic skills of students with individual mild difficulties to the typically developing students and found that almost 80% of students had poor pragmatic language skills [22].

Of relevance to this study is recent research which has shown that assessment and diagnosis play a vital role in ensuring typical language development for preschool children [23]. Because PLD includes multidimensional skills such as higher cognitive skills (e.g., inference, theory of mind) [24], the early identification of atypical language development, including PLD, is essential. This could include, for instance, preterm children who could be assessed early to ensure typical language development moving to the grade school level [25]. The assessment of PLD and diagnosis of PLI are also advantageous in atypical PLD found in children who are deaf or hard of hearing [26]. In many cases, this leads to early intervention and training to bridge gaps, in oral language skills, for instance, between preschool children from low socioeconomic status and others [27]. Although socioeconomic status and bilingual exposure relate to preschool children’s linguistic skills, including PLD, this relationship remains independent [28]. It is worth considering other influential factors underlying children’s communication difficulties, such as emotional competences [29] and social cognition [30].

### Purpose of the Present Study

There is a lack of literature examining the development of pragmatic skills in Arabic literature. However, a few studies have attempted to explore some aspects of pragmatic skills development in preschool children and children in elementary school with impaired and normal abilities. For instance, in a study, the researchers compared the results of the Arabic Test of Pragmatic Language (TOPL-2)—as a psycholinguistic marker that measures the ability of individuals with Developmental Dysphasia (DD)—and the results of both the Pragmatics Profile (PP) and Observational Rating Scale ORS subtests from the Clinical Evaluation of Language Fundamentals (CELF-4) [31]. In another study, the researchers attempted to design a test to standardise an Egyptian Arabic Pragmatic Language Test (EAPLT) using linguistic and social questions and pictures to address specific deficit aspects in this language domain [32]. As such, the present study contributes to validating a version of the PLSI in Arabic for assessing PLD in school settings and diagnosing PLI in clinical settings.

## 2. Method

### 2.1. Sample

The theoretical population of this study was preschoolers who speak the Arabic language as their mother tongue language, with and without a psychiatric history. The accessible population was preschoolers in Saudi Arabia with and without a psychiatric history. The sampling frame included preschoolers who were enrolled or not enrolled in preschools in Saudi Arabia. We defined preschoolers here as children who had not joined basic education, which could have been ≤7.0 years. The sample included 237 preschoolers without pragmatic language impairment and 27 with pragmatic language impairment. A detailed description of the population is provided in Table 1 and Table 2.

A total of 237 preschoolers between 4 and 7 years, both females and males, in different areas in Saudi Arabia were randomly selected for participation in the validation of the Arabic version of the PLSI. A detailed description of the participants is provided in Table 2.

A total of 27 Arabic-speaking Saudi children with various communication abilities were selected to assess the application of the A-PLSI at the Jeddah Institute for Speech and Hearing and Medical Rehabilitation (JISH), Jeddah, Saudi Arabia. JISH is a clinic that provides assessment and treatment for children and adults with various communication disorders. Amongst the participants, twenty children had different neurodevelopmental disorders; the remaining seven did not have any concomitant disorders. Informed consent was signed by all parents of children involved in this study. The Research Committee also approved the study at JISH. Table 1 shows the characteristics of participants in the clinical setting.

### 2.2. Instrument

The PLSI is designed to assess children’s pragmatic language skills [20]. Theoretically, the instrument is designed on the theoretical bases of pragmatics [33,34,35]. The authors of the instruments used the rules of communication introduced by Bates [34]. These include: (1) “corporate with your conversational partner”; (2) “tell the truth”; (3) “consider maxims of speech (quality, quantity, relevance, and manner)”; (4) “request only information you sincerely want to have”; (5) “give your listener just the right amount of background information”; (6) “be unambiguous”; and (7) “change your language to fit each current social situation” [20] (pp. 1,2).

The PLSI is introduced in 45 items divided into three subscales: classroom interaction (CI), social interaction (SI), and personal interaction (PI). The use of the instrument includes: (1) identifying students who have PLI; (2) documenting progress in pragmatic language ability; (3) determining strengths and weaknesses in pragmatic language skills; and (4) data-collection for research [20].

The translation process went through several stages before reaching the A-PLSI. Empirical evidence for the validation process is presented in detail in the results. We outline the main steps here. First, the instrument was translated using the literal translation by two academics holding doctoral degrees in translation and curriculum design. Back translation was conducted to ensure the accuracy of the content. The first draft was shared with three academics majoring in clinical linguistics, psycholinguistics, and speech–language pathology. Modifications and suggestions were provided for the further development of the translation, but none of these modifications were related to cultural differences. These suggestions are provided in Table 3. Having agreed to the final draft of the translation, the instrument was administered to (n = 30) cases for piloting purposes. The first author reviewed the results and ensured the accuracy of the collected data.

All 45 items of the test were administered; however, some items were modified as they were not fully applicable in the clinical setting. These modifications included items that required reading or writing or were classroom specific. For example, Item 13, ‘writing a good story’, was modified into ‘telling a complete story’. The complete list of modifications is described in Table 4.

The English PLSI was normed on 1175 children between 5 and 12 in different areas of the United States. The data were collected between 2001 and 2004. The authors mentioned that they included additional data for children with disabilities. Coefficient alpha, test–retest, and interrater reliabilities were reported for this instrument. Content validity, item discrimination, and criterion-related validities were also reported. The validity also included construction validity and factor analysis. The three subscales and the pragmatic language index achieved acceptable values, confirming the measurability of the instrument for its sought purposes. We located only one attempt to validate the PLSI in the Turkish language [21]. The participants included 1383 children between 5 and 12 years with typical (language) development in different areas of Turkey. The authors collected additional data from children with intellectual disabilities and autism and reported that the Turkish version of the PLSI could discriminate between the two types of children and assess pragmatic language skills in both cases [21]. The authors reported that they made some social and/or cultural modifications while translating the instrument. They also reported that this instrument remains insufficient to make concrete decisions on the level of pragmatic language development in children due to the interdisciplinary nature of pragmatic language skills.

### 2.3. Design

Since the purpose of the study was to validate the Arabic version of the PLSI, it was vital to compare data from children with and without a psychiatric history. All participants in the two groups were assigned randomly. Although the participants in the clinical group were selected to match the age requirement, no limitation for the type of disorder or even IQ level was considered.

### 2.4. Procedures

The data were collected between 19 October 2021 and 13 January 2022. Preschool teachers administered the instrument in the randomly selected schools in Saudi Arabia (See Table 2). The administration time for each participant was between 5 and 10 min. The preschool teachers were trained by the third and fourth authors, who were trained by the first author, to administer the test. The teachers filled in the required information based on their knowledge and experience of spending time with their students. An institutional review board (IRB) was obtained from King Saud University, Saudi Arabia for the data collection from preschools. All participants included in this study were reported as not being enrolled in basic education regardless of their age at data collection.

Participants who met the criteria of this study were included regardless of the severity or type of communication disorder or the amount of time spent in therapy. Following the participants’ selection, the speech–language pathologist (SLP) administered the test and provided them with therapy. Pragmatics and social skills goals were always incorporated within any patient’s treatment plan. However, more goals targeting specific areas of weakness were included in the treatment plans for children who had affected social skills or were diagnosed with ASD. Various approaches were utilised to achieve those goals, such as social scripts, social stories, and social groups to generalise skills. Parents were also an integral part of therapy as all treatment plans were family-oriented. The parents were included in the therapy sessions to transfer learnt skills to the home environment.

The data analysis went through several steps. First, all the data were moved from the booklets to Excel sheets. The Excel sheets were checked to ensure data accuracy. The Excel sheet was translated into English since the original one was made in Arabic. The data were then analysed using Minitab 18 and Jamovi 2.2.2. Both descriptive and inferential tools were used to analyse the collected data and achieve the study’s objective. The results are reported in detail in the following section.

## 3. Results

The objective of this study was to provide evidence of the validity of the A-PLSI. First, we present psychometric evidence for the validation of the instrument. This is explained in three sub-sections: normative information, validity, and the reliability of the A-PLSI. Second, we present empirical evidence demonstrating the instrument’s usability to assess PLD in a school setting and diagnose PLI in a clinical setting. This sub-section, which presents the characteristics of PLD in preschool children, is demonstrated in three parts: PLD and gender, PLD and pragmatic language skills (CI, SI, and PI), and PLD and age.

### 3.1. Normative Information

The A-PLSI was normed on 264 children between the ages of 4.0 and 7.0 years residing in 15 cities in Saudi Arabia: Al Jubail, Al Khobar, Al-Kharj, AlNamas, Altaif, Eastern region, Hafar Al-Batin, Hail, Jeddah, Khamis Mushait, Makkah, Medina, Riyadh, Taif, and Yanbu.

The data were collected between 2021 and 2022. Preschools and kindergartens within the aforementioned cities were randomly selected to participate in this study. Those who had special needs all resided in Jeddah. We focused on preschoolers during this study stage, but we plan to extend to children aged 5 to 12. The raters included preschool teachers and speech–language clinicians. A description of the representativeness of the normative sample is given below in Table 2.

### 3.2. PLSI-A Validity

#### 3.2.1. Construct Validity

For establishing the validity of the Arabic version of PLSI, we considered both face validity and content validity. Below, we describe the procedures we used to establish construct validity.

Face Validity: To establish face validity, we followed two steps. In the first instance, the first author reviewed the translation and determined its relevance to the original. Secondly, the translation was sent to four experts in speech–language pathology, psycholinguistics, translation, and curriculum design. Based on feedback, the A-PLSI appeared to be suitable for evaluating PLD. A few items regarding translation and age-matching concerned the speech–language pathologist. First, Item 13 on the classroom interaction subscale (writing a good story) was considered unsuitable for preschoolers. This was discussed, and it was decided that advanced preschoolers could accomplish this, and it is typical of preschoolers to get involved in short storytelling. The second item was 20 in the social interaction subscale, where it was stated that taking turns in conversations was different from conversing. The proposed translation was used in place of the previous one. The other items are listed in Table 4.

Content Validity. This scale was designed to validate the validated scale in English, so we compared the validity of the content to the three subscales already included in the original version. The PLSI is composed of three main subscales: classroom interaction (Items 1–15), social interaction (Items 16–30) and personal interaction (Items 31–45). Teachers and speech–language pathologists who administered the scale confirmed that these three subscales were appropriate for measuring PLD in preschoolers.

#### 3.2.2. Criterion-Related Validity

Predictive Validity: PLD scores were correlated with preschoolers’ performance on CI, SI, and PI, assuming that A-PLSI would predict PLD level for preschoolers (See Table 3). PLD and CI, SI, and PI were significantly correlated as r = 0.95, *p* = 0.001, r = 0.90, and *p* = 0.001, respectively. A high correlation score indicated that the A-PLSI correctly predicted PLD for preschoolers through CI, SI, and PI.

**Figure 1:**
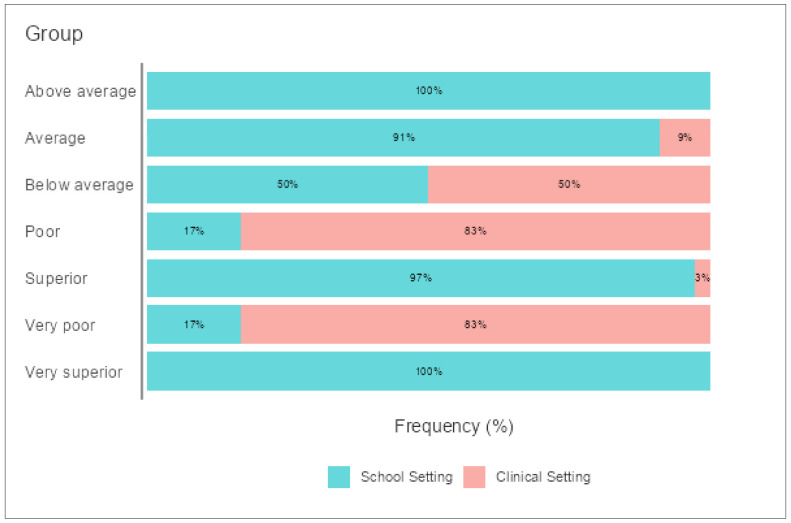
Comparing performance of participants using the Pragmatic Language Index of A-PLSI.

Concurrent Validity: To test the ability of the scale to distinguish between preschoolers with and without psychiatric histories, we compared the results of two groups of preschoolers. A one-way between-subjects ANOVA was conducted to compare preschoolers in schools and clinical settings in terms of PLD represented in three dimensions (CI, SI, and PI) (See Table 5). There was a significant effect of PLI at the *p* < 0.05 level for the three dimensions: F (114, 35) = 1.30, *p* = < 0.001; F (69, 56) = 1.29, *p* = < 0.001; and F (45, 93) = 1.29, *p* = < 0.001, respectively. This significance was also reported for the overall PLD and pragmatic language index: F (80, 84) = 1.29, *p* = < 0.001 and F (67, 81) = 1.29, *p* = < 0.001. Post hoc comparisons using the Games–Howell test indicated that the means between these two groups for all dimensions were statistically significant *p* = < 0.001 (see Table 3 for means and standard deviations). Taken together, these results suggest that the presence of any disorder influences PLD in CI, SI, PI, or overall development. Specifically, our results suggest that preschoolers who show any signs of atypical development will experience a delay in their PLD. Figure 1 illustrates the performance of the two groups in these three dimensions, distributed according to their group setting and level of pragmatic language skills.

### 3.3. Factor Analysis

To assess the data structure and further check the validity of the A-PLSI, we evaluated the correlations between the variables by factor analysis using Minitab 18 (See Table 6). This was accomplished in three steps. First, we determined the number of factors using the maximum likelihood factor analysis of the correlation matrix in two ways: unrotated and varimax rotation. Next, we interpreted the factors and checked for data problems.

**Figure 2:**
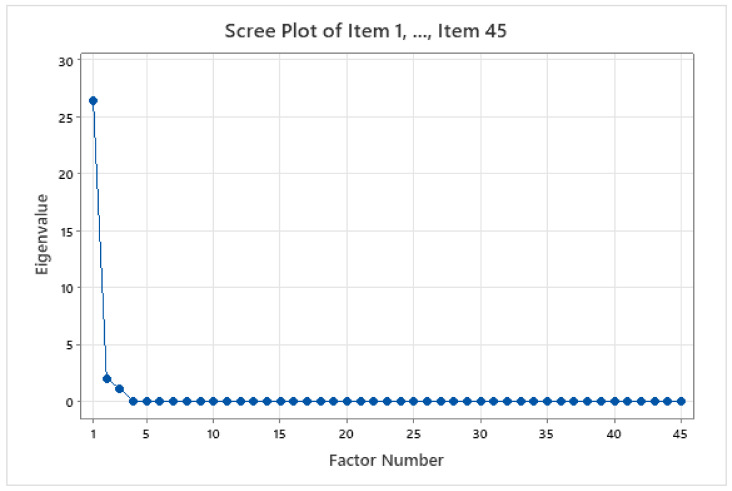
Score plot for Items 1–45 of the A-PLSI.

**Figure 3:**
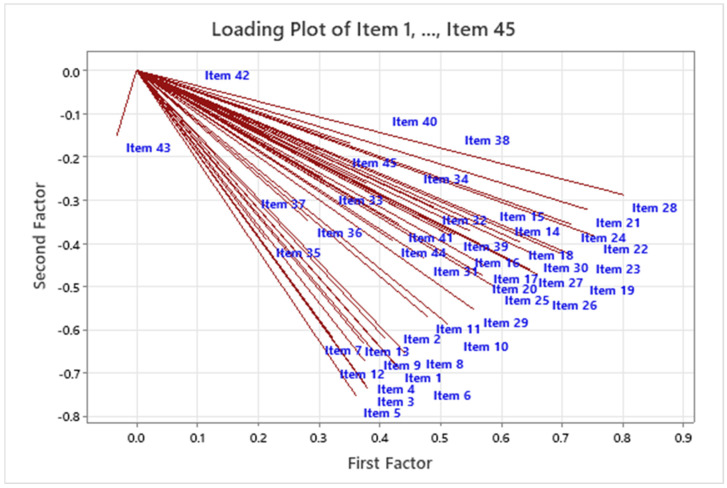
Loading plot for Items 1–45 of the first and second factors for the A-PLSI. Note: This loading plot visually illustrates the loading results for the first two factors.

**Figure 4:**
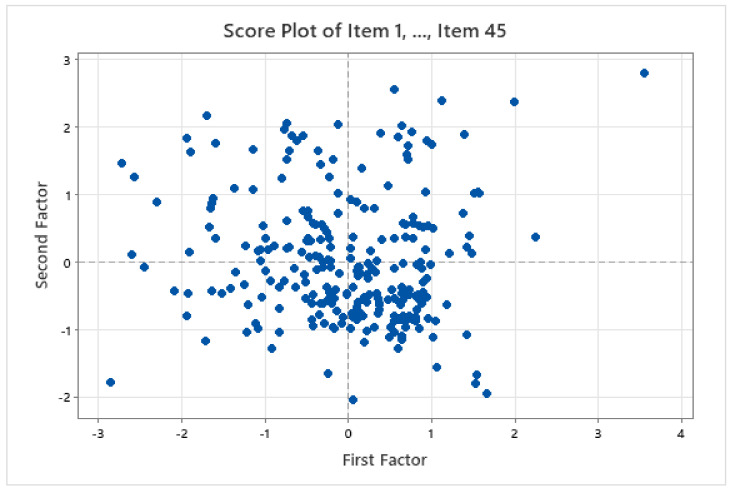
Score plot for Items 1–45 of the first and second factors for the A-PLSI. Note: As can be seen from the score plot, the data appear normal, and no extreme outliers are apparent except for the data values shown on the upper right and lower left sides of the plot, which are further away from the other data points.

These results show the unrotated factor loadings for all the factors using the maximum likelihood extraction method. This method was utilised because the scale had already been identified with the three factors used to measure PLD. The three factors had variances (eigenvalues) that were greater than 1. The percentage of variability explained by factor 1 was 0.586. Factors 2 and 3 explained the percentage of variability of 0.044 and 0.026. Figure 2 illustrates that these three factors contributed most to the variability in the data. The remaining factors accounted for a very small proportion of the variability and were likely unimportant. Figure 3 shows the loading plot for the first two factors, which look consistent except for Item 43. Figure 4 is a scope plot showing the distribution of items for the first factor, indicating normal distribution.

In comparison, these results performed a varimax rotation on the data in the second part of the table to the right. Items 21–24 (0.741, 0.753, 0.713, 0.716) had large positive loadings on factor 1; this factor described classroom interaction and the potential for developing pragmatic language skills. Items 3–5 (−0.737, −0.726, −0.753) had large positive loadings on factor 2; this factor described social interaction and the potential for developing pragmatic language skills. Items 33–34 and 37 (0.806, 0.800, 0.725) had large positive loadings on factor 3; this factor described personal interaction and the potential for developing pragmatic language skills. Together, all three factors explained 0.655 of the variation in the data.

### 3.4. Confirmatory Factor Analysis

**Figure 5:**
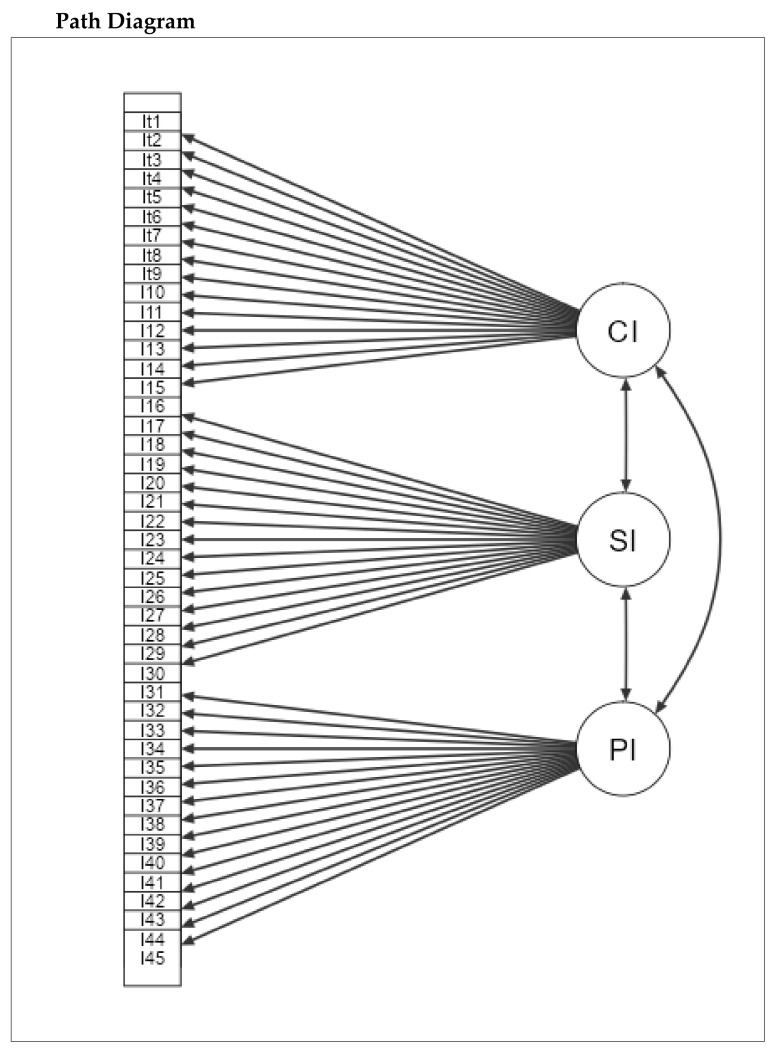
Path diagram for the subscales of A-PLSI.

To verify our previous steps for the validity of the scale to measure PLD on preschoolers with and without PLI, a CFA was performed using Jamovi 2.2.2. First, we checked the model fit, and it was satisfactory to run CFA (*p* < 0.001). Second, we checked the fit of measures where the CFI and TLI reported high values (0.814, 805) with a low RMSEA (0.099) and a 95% CI (0.095, 0.103). This also confirmed that the measure was fit to run this analysis. Table 7 shows the factor loadings; all the *p*-values were significant (*p* < 0.001) and had standard estimates (>40). More importantly, the factor covariances for the three subscales CI, SI and PI were all significant (*p* < 0.001) with high standard estimates (0.914, 0.828, 0.833). Finally, the path diagram (see Figure 5) confirmed that the three factors were associated with each, and the items of each factor were fit to one another. This analysis indicated that the data matched our hypothesised structure for the proposed three factors regarding the measurement of PLD through CI, SI and PI.

### 3.5. PLSI-A Reliability

#### Internal Consistency Reliability

Reliability was established using Cronbach’s Alpha (See Table 8). The PLSI-A was highly reliable (45 items; α = 0.98). Each of these items was also highly reliable (α = 0.98).

**Figure 6:**
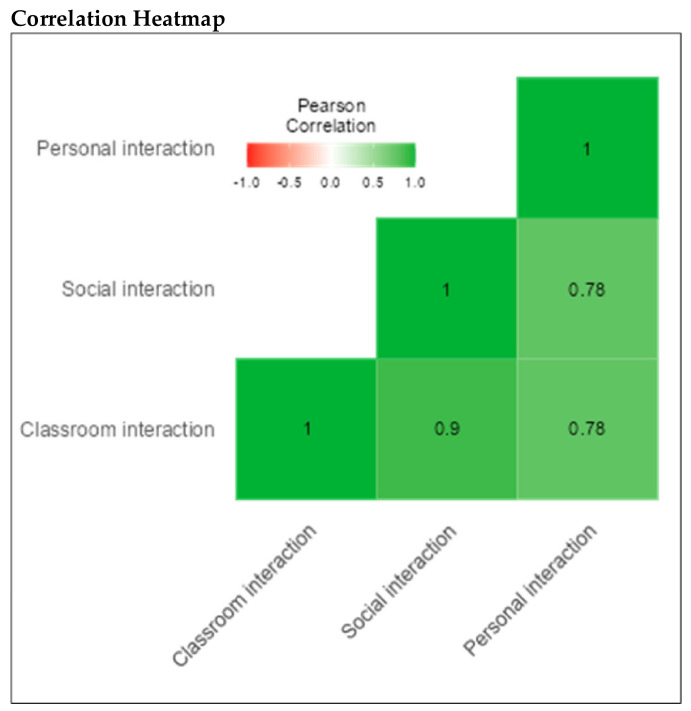
Correlation heatmap for subscales.

The CI subscale consisted of 15 items (α = 0.88), the SI subscale consisted of 15 items (α = 0.87), and the PI subscale consisted of 15 items (α = 0.94). Cronbach’s alphas for the 15 CI, 15 SI and 15 PI items was 0.93 (See Table 9). These positive correlations are further illustrated in Figure 6.

**Figure 7:**
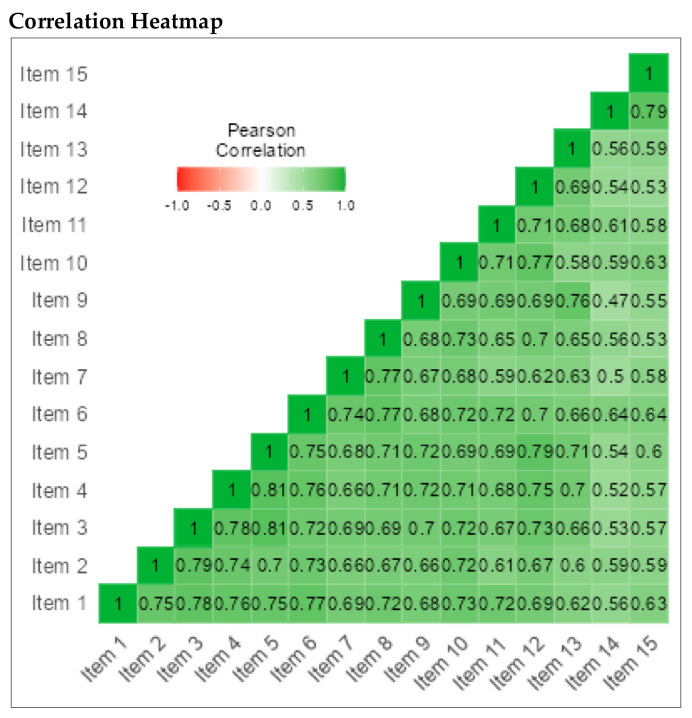
Correlation heatmap for IC subscale; 15 items.

**Figure 8:**
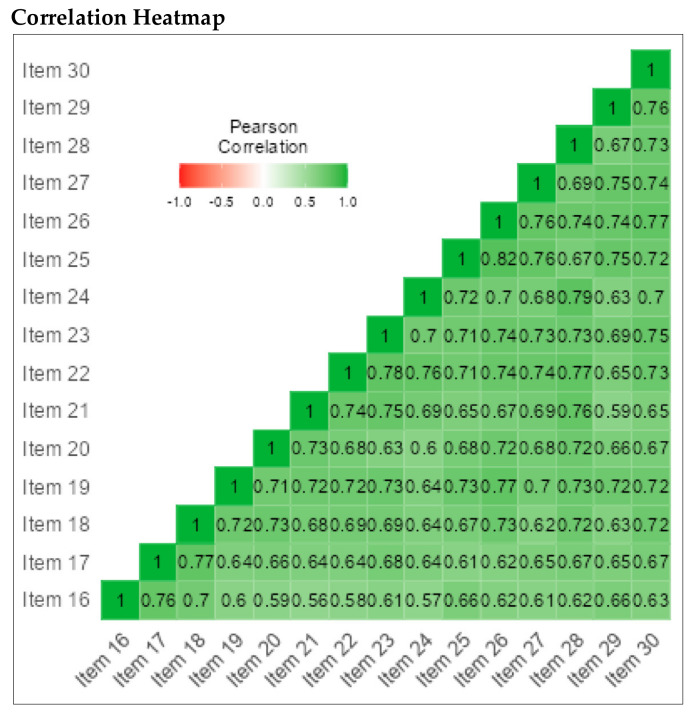
Correlation heatmap for IS subscale; 15 Items.

**Figure 9:**
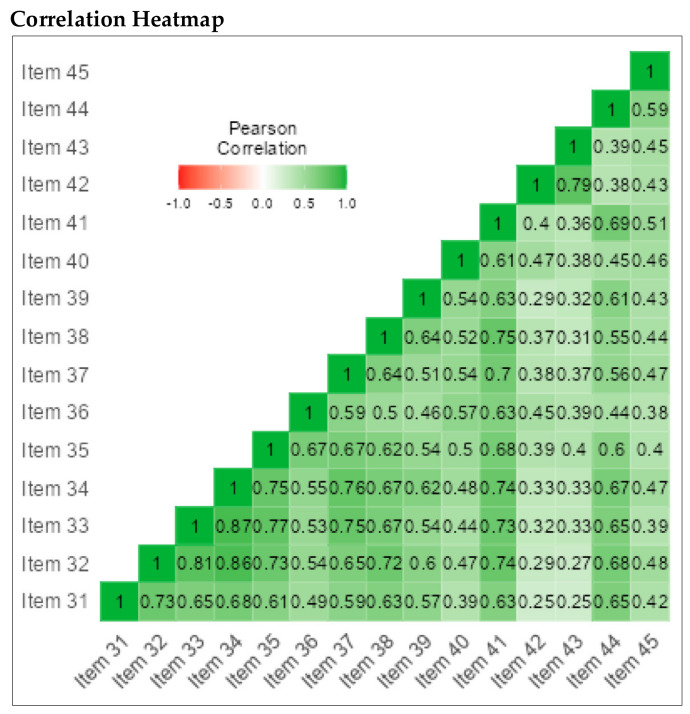
Correlation heatmap for IP subscale; 15 Items.

We also conducted reliability analyses for each subscale of the PLSI-A separately. The CI subscale consisted of 15 items (α = 0.97), the SI subscale consisted of 15 items (α = 0.97), and the PI subscale consisted of 15 items (α = 0.94) (See Figure 7, Figure 8 and Figure 9).

### 3.6. Characteristics of PLD in Preschoolers Using the A-PLSI

A one-way ANOVA was performed to determine if the proportion of PLD represented by CI, SI and PI differed by gender, PLS, or age.

#### PLD and Gender

**Figure 10:**
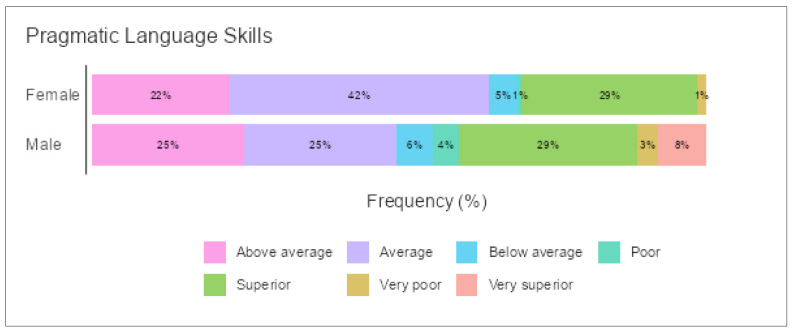
Pragmatic language skills according to gender for all participants in the A-PLSI.

A main effect of gender was found for CI: F (4, 61) = 1.229, *p* < 0.033; SI: F (7, 102) = 1.217, *p* < 0.008; and PI: F (6, 68) = 1.227, *p* < 0.010. A post hoc comparison using Games–Howell was performed to verify this statistical significance, and the results indicated differences among preschoolers in the three dimensions of PLD according to gender. Generally, females showed higher PLD skills in CI, SI, and PI. There was also a main effect in the total performance of pragmatic language skills: F (6, 85) = 1.224, *p* < 0.009. Females reported significantly higher scores than males (see Table 10 for means and standard deviations). Figure 10 shows preschoolers’ performance in these three dimensions distributed by gender and grade of pragmatic language skills.

The analysis of the data revealed an issue regarding the influence of score conversion on participants’ overall performance based on gender. There was a difference conversion sheet provided for scores based on gender by the authors of the PLSI. For females, the conversion scores were lower when compared with males, who were given higher conversion scores when raw scores were converted to percentiles and standard scores. Females generally showed higher PLD scores than males, but this was completely reversed when converted scores were considered. Initially, all *p*-values were insignificant (*p* > 0.0). The significance of these differences was also tested using the post hoc comparison of Games–Howell. There was only significance for the standard score of SI *p* > 0.021. While the means of these conversions showed differences favouring males, these differences were statistically insignificant except for the SI. The reported differences for the raw scores were more valid, especially when looking at the pragmatic language index (M = 110) for females and (M = 111) for males.

### 3.7. Pragmatic Language Development and Pragmatic Language Skills

**Figure 11:**
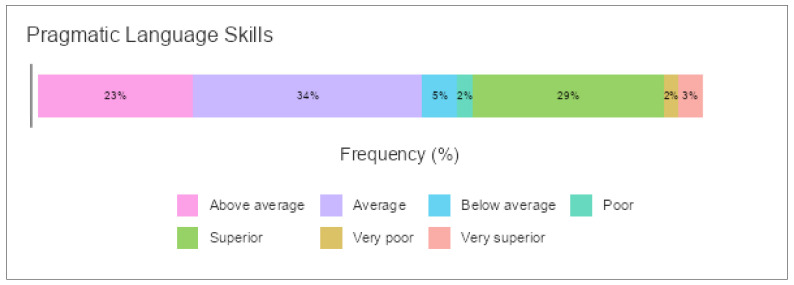
Pragmatic language development according to pragmatic language skills in the A-PLSI.

A main effect of gender was found for CI: F (417) = 6.29, *p* < 0.001; SI: F (219) = 6.29, *p* < 0.001; and PI: F (376) = 6.31, *p* < 0.001. A post hoc comparison using Games–Howell was performed to verify this statistical significance, and the results indicated differences among preschoolers in the three dimensions of PLD according to the grade of pragmatic languages except for a few items. For instance, there was no significant difference between ‘below average’ and ‘poor’, ‘poor’ and ‘very poor’, or ‘superior’ and ‘very superior’ in the case of CI. This also applied to the case of SI and PI. In general, the highest means were reported for the ‘very superior’ grade showing a high level of PLD for preschoolers (see Table 11 for means and standard deviations). Figure 11 shows preschoolers’ performance in these three dimensions distributed by grade of pragmatic language skills.

#### Pragmatic Language Development and Age

**Figure 12:**
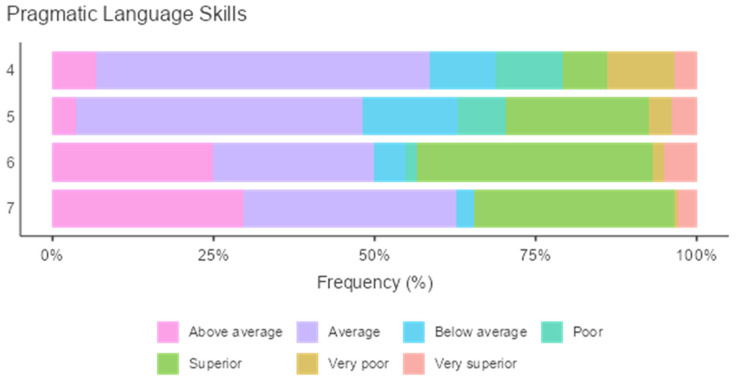

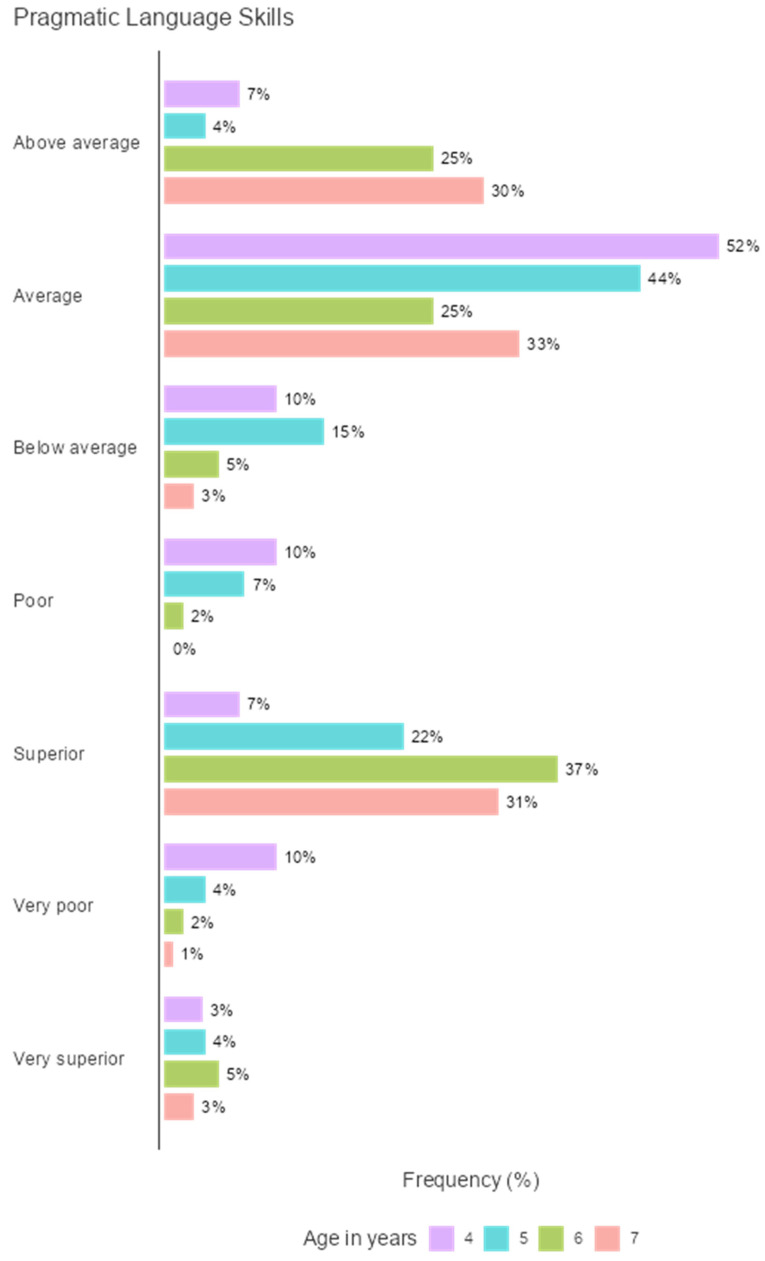
Performance of preschoolers in CI, SI and PI distributed by age in years.

A main effect of age in years was found for CI: F (14.71) = 3.64, *p* < 0.001; SI: F (16.90) = 3.64, *p* < 0.001; and PI: F (6.74) = 3.64, *p* < 0.001. A post hoc comparison using Games–Howell was performed to verify this statistical significance, and the results indicated differences among preschoolers in the three dimensions of PLD according to the participants’ age, except for a few items. For instance, there was no significant difference between ages 4 to 5, 5 to 6, and 6 to 7 in the case of CI and SI. More importantly, there was only reported significance for ages 4 to 6 and 4 to 7 in the case of the third dimension, that is, PI. In general, the highest means were reported for ages 6 and 7, showing a high level of PLD for preschoolers (see Table 12 for means and standard deviations). Figure 12 shows preschoolers’ performance in these three dimensions distributed by age.

## 4. Discussion

This study aimed to provide empirical evidence for the psychometric features of a validated version of the PLSI. This purpose was tested through the possible identification of preschool children with PLI, documenting the progress of pragmatic language ability, and comparing the characteristics of PLD between preschooler children with and without psychiatric histories. The results were presented in two sections. The first section presented the psychometric features for the A-PLSI, including normative information, validity, and reliability. The second included empirical evidence for the ability of the A-PLSI to measure PLD in preschoolers and identify differences among them considering age, gender, and pragmatic language skills. There are two key findings of the present research. First, the method showed acceptable psychometric features as an assessment instrument for measuring PLD in preschool children. The evidence showed that preschoolers without PLI outperformed those with PLI in CI, SI, PI, and overall PLD. Second, the A-PLSI could document pragmatic skills in preschooler children considering CI, SI, and PI and distinguish between children with and without PLI.

The first key finding can be further extended into three findings. First, the A-PLSI is a representative, normed instrument. It was normed on 264 preschool children in Saudi Arabia distributed in different cities, including females and males and populations with and without a psychiatric history. Second, a high level of validity was established for the A-PLSI. This was achieved by considering both construct validity and criterion-related validity. The construct validity was achieved through face validity by different raters, and content validity by dividing the whole scale into three subscales—CI, SI, and PI—to measure PLD. Criterion-related validity was achieved through predictive validity, concurrent validity, factor analysis, and confirmatory factor analysis. All these reported a high level of validity, making A-PLSI a valid instrument with acceptable psychometric features that can assess PLD and diagnose preschooler children with and without PLI. Third, internal reliability was measured, and the results reported that the A-PLSI was highly reliable (45 items; α = 0.98).

The second finding can be extended into three findings. First, the A-PLSI reported controversial differences between females and males in PLD considering CI, SI and PI. When the results were calculated using the direct data, females reported higher PLD in CI, SI and PI. When the data were converted using the pragmatic language index provided by the authors of PLSI, males reported higher scores than females, albeit statistically insignificantly, except in SI. Second, the A-PLSI reported different levels of PLD for the participants according to three skills, namely, CI, SI and PI, with different grades including (very) superior, (above/below) average, and (very) poor. Third, the A-PLSI showed higher PLD for children with older age, that is, 6 and 7 years, compared to 4 and 5, who showed lower levels of PLD.

This pattern of results is consistent with the previous literature reporting the need to validate or construct instruments for assessing PLD and the diagnosis of PLI in the Arabic language. Among the instruments validated were TOPL-2 and PP and ORS from CELF-4. The TOPL-2 is a formal assessment tool based on tasks to assess PLD in children with and without PLI. The PP and ORS are informal instruments used for the same purpose but filled in by parents of the children. They make a credible combination, allowing a triangular assessment of PLD and diagnosis of PLI [31,32,36,37,38,39,40]. The results are also consistent with the claim that the PLSI is a useful instrument for assessing PLD in both school and clinical settings, but it remains insufficient to make final decisions on the existence of PLI or required rehabilitation programs. In other words, there is a need to accompany the use of these instruments with others to reach a better decision about the assessment of PLD and the diagnosis of PLI [21].

### 4.1. Implications for Practice

#### 4.1.1. Validation of Assessment Tools for Pragmatic Language Development

These data have some potential practical implications. For example, instruments for assessing PLD in Arabic are scarcely available, which might motivate other researchers interested in research in Arab countries to follow similar steps. Previous efforts have been made to validate or construct instruments. These include the validation of the Test of Pragmatic Language (TOPL-2), the Pragmatics Profile and Observational Rating Scale subtests from the Clinical Evaluation of Language Fundamentals (CELF-4) using modern standard Arabic [39,41]; LUI [42] by [40]; and the developed instrument of the Egyptian Arabic Pragmatic Language Test (EAPLT) [32].

#### 4.1.2. Early Diagnosis of Pragmatic Language Impairment

There are no accurate incidence reports (i.e., number of newly identified cases of PLI) or prevalence (i.e., number of children with PLI) available but there are generally higher rates among children with developmental language disorders, particularly boys [43]. Our findings highlight that PLI often creates many challenges for (preschool) children. Depending on the severity of the disorder, these challenges include difficulties such as making and maintaining friendships, isolation and poor peer acceptance, and difficulties integrating with society. The early identification of pragmatic language disorders is an integral part of minimising these challenges and providing the child with the tools needed to have appropriate social interactions.

### 4.2. Limitations

There are at least two potential limitations concerning the results of this study. The first limitation concerns the number of included participants. While we intended to include a higher number of preschool children in both school and clinical settings, there was much hesitation from schools and clinics to allow accessibility due to the restrictions concerning COVID-19. A second potential limitation, also related to the pandemic, is that it was not possible to administer another test to compare the results of the A-PLSI.

## 5. Conclusions

The findings of this study revealed that the A-PLSI is a valid instrument that can be used to identify children with and without PLI in Arabian contexts. The presented evidence illustrated that the established psychometric features for the A-PLSI could be reliably used to measure PLD concerning CI, SI, and PI to document the strengths and weaknesses of children in terms of pragmatic language ability. Furthermore, the presented evidence confirms that the A-PLSI could be used to measure typical PLD according to age, and it was shown that the performance of children increased according to their age. While these results are positive indicators that this instrument could be used by other researchers, it is vital to strengthen this method with other ones. The data could be collected twice by teachers, parents, and speech–language clinicians for validation. Another method is to use multiple instruments that could be formal, informal, or observational to reach a more concrete assessment, especially when used for diagnostic purposes in clinical settings or for the early identification of children vulnerable to atypical pragmatic language development.

## Figures and Tables

**Table 1:** Characteristics of participants in the clinical setting.

Variable	Characteristics
No. of participants	27
Gender	F: 5; M: 22
Age Range	4–6
Communication Disorders	SLD: 22; LD: 4; stuttering: 1
Concomitant disorders	ADHD: 3; ASD: 5; AOS: 4; HI: 3; DD: 1; DS: 1; AOS/Dyslexia: 1; ADD/DD: 1
Language	Arabic
Nationality	Saudis

SLD—speech and language delay; LD—language delay; ADHD—attention deficit hyperactivity disorder; ASD—autism spectrum disorder; AOS—apraxia of speech; HI—hearing impairment; DD—developmental delay; ADD—attention deficit disorder.

**Table 2:** Respondent characteristics.

	School Setting (N)	Clinical Setting (N)	%	
**Age Group**	237	27		
4	14	15	6	55
5	19	8	8	30
6	56	40	24	150
7	148	0	62	0
**Gender Group**				
Female	142	5	60	19
Male	95	22	40	81
**City Group**				
Riyadh	158		67	
Eastern region	18		8	
Jeddah	14	27	6	100
Khamis Mushait	14		6	
Makkah	10		4	
Other cities	23		9	
**Socioeconomic Status**				
*Father employment*				
Employed	227	23	96	85
Unemployed	10	4	4	15
*Mother employment*				
Employed	127	2	54	7
Unemployed	110	25	46	93
*Father education*				
Middle school	6	0	3	0
Secondary school	58	7	24	26
Bachelor’s degree	141	13	59	48
Master’s degree	17	4	7	15
Doctorate	15	3	6	11
*Mother education*				
Middle school	15	1	6	4
Secondary school	44	7	19	26
Bachelor’s degree	150	16	63	59
Master’s degree	23	2	10	7
Doctorate	5	1	2	4
**Exceptionality Status**				
No exceptionality	237	27	NA	NA
Attention deficit hyperactivity disorder	NA	3	11	NA
Hearing impairment	NA	3	11	NA
(Speech and) language delay	NA	10	37	NA
Childhood apraxia/dyslexia	NA	5	18.5	NA
Autism spectrum disorder	NA	4	15	NA
Developmental delay	NA	1	3.5	NA
Down’s syndrome	NA	1	3.5	NA

The sample consisted of 264 preschoolers (M = 6.24, SD = 1.02). There were two groups of participants: school settings (N = 237, M = 6.43, SD = 0.873) and clinical settings (N = 27, M = 4.59, SD = 0.747).

**Table 3:** Predictive validity values for A-PLSI and its subscales.

		Pragmatic Language Development	Classroom Interaction	Social Interaction	Personal Interaction
Pragmatic Language Development	Pearson’s r	—			
	*p*-value	—			
Classroom interaction	Pearson’s r	0.955 ***	—		
	*p*-value	<0.001	—		
Social interaction	Pearson’s r	0.952 ***	0.896 ***	—	
	*p*-value	<0.001	<0.001	—	
Personal interaction	Pearson’s r	0.906 ***	0.782 ***	0.782 ***	—
	*p*-value	<0.001	<0.001	<0.001	—

Note. *** *p* < 0.001.

**Table 4:** Sample Corrections and Modifications for A-PLSI.

Item No.	Suggestion/Correction/Translation in English	Arabic Correction Sample
Item (8)	Correcting *Linguistic* mistakes: subject verb agreement	تصحيح الأخطاء *اللغوية* فور إدراكها (مثال: هو تاكل .. هو ياكل)
Item (9)	Giving an oral book report: *Retelling a complete story*	*إعادة سرد قصة مع ذكر جميع الأحداث*
Item (11)	Getting the meaning of texts that explain how something works: *Ability to sequence events in the correct order receptively*	*ترتيب خطوات حدث معين بشكل صحيح*
Item (12)	Explaining how things work: *sequencing events verbally*	*التعبير لفظيا عن خطوات حدث معين بشكل صحيح*
Item (13)	Writing a good story: *Telling a story*	سرد قصة بشكل صحيح

**Table 5:** Establishing concurrent validity for the A-PLSI and its subscales.

Variable	Group	N	Mean	SD	SE
Classroom interaction	School Setting	237	104.32	22.35	1.452
	Clinical Setting	27	49.81	25.39	4.886
Social interaction	School Setting	237	108.02	21.17	1.375
	Clinical Setting	27	62.33	27.55	5.303
Personal interaction	School Setting	237	102.64	21.22	1.378
	Clinical Setting	27	64.30	28.51	5.487
Pragmatic Language Development	School Setting	237	314.98	58.86	3.824
	Clinical Setting	27	176.44	77.56	14.926
Pragmatic Language Index	School Setting	237	113.42	13.06	0.849
	Clinical Setting	27	86.26	16.56	3.188

**Table 6:** Maximum likelihood factor analysis of the correlation matrix.

Unrotated Factor Loadings and Communalities					Rotated			
Item	Factor 1	Factor 2	Factor 3	Com.	Factor 1	Factor 2	Factor 3	Com.
Item 1	0.818	−0.156	−0.197	0.733	0.427	−0.683	0.291	0.733
Item 2	0.802	−0.074	−0.168	0.677	0.409	−0.621	0.352	0.677
Item 3	0.819	−0.166	−0.268	0.770	0.380	−0.737	0.286	0.770
Item 4	0.825	−0.133	−0.265	0.768	0.376	−0.726	0.317	0.768
Item 5	0.822	−0.155	−0.294	0.786	0.361	−0.753	0.298	0.786
Item 6	0.820	−0.166	−0.199	0.740	0.430	−0.689	0.284	0.740
Item 7	0.780	0.010	−0.231	0.661	0.323	−0.620	0.415	0.661
Item 8	0.809	−0.154	−0.166	0.705	0.441	−0.654	0.287	0.705
Item 9	0.796	−0.103	−0.202	0.684	0.391	−0.652	0.326	0.684
Item 10	0.838	−0.109	−0.072	0.719	0.511	−0.587	0.336	0.719
Item 11	0.797	−0.108	−0.082	0.653	0.478	−0.570	0.317	0.653
Item 12	0.811	−0.081	−0.229	0.717	0.375	−0.672	0.353	0.717
Item 13	0.755	−0.120	−0.199	0.624	0.374	−0.633	0.290	0.624
Item 14	0.692	−0.054	0.077	0.488	0.506	−0.374	0.303	0.488
Item 15	0.743	0.001	0.082	0.558	0.522	−0.380	0.376	0.558
Item 16	0.748	0.011	0.076	0.565	0.518	−0.383	0.388	0.565
Item 17	0.782	−0.103	0.068	0.627	0.573	−0.452	0.307	0.627
Item 18	0.829	−0.028	0.144	0.708	0.630	−0.397	0.392	0.708
Item 19	0.803	−0.199	0.179	0.716	0.696	−0.423	0.231	0.716
Item 20	0.831	−0.048	0.047	0.695	0.569	−0.476	0.381	0.695
Item 21	0.770	−0.180	0.282	0.705	0.741	−0.322	0.227	0.705
Item 22	0.795	−0.239	0.249	0.751	0.753	−0.384	0.190	0.751
Item 23	0.809	−0.216	0.189	0.737	0.713	−0.425	0.219	0.737
Item 24	0.776	−0.182	0.240	0.693	0.716	−0.357	0.230	0.693
Item 25	0.850	−0.081	0.043	0.731	0.590	−0.502	0.362	0.731
Item 26	0.856	−0.131	0.114	0.763	0.660	−0.474	0.320	0.763
Item 27	0.804	−0.182	0.117	0.693	0.647	−0.461	0.249	0.693
Item 28	0.807	−0.153	0.347	0.795	0.801	−0.288	0.265	0.795
Item 29	0.811	−0.171	−0.015	0.687	0.555	−0.555	0.267	0.687
Item 30	0.826	−0.109	0.143	0.715	0.655	−0.427	0.322	0.715
Item 31	0.800	0.090	0.003	0.649	0.473	−0.435	0.485	0.649
Item 32	0.841	0.299	0.090	0.804	0.489	−0.317	0.682	0.804
Item 33	0.769	0.481	−0.003	0.823	0.317	−0.271	0.806	0.823
Item 34	0.798	0.460	0.047	0.851	0.377	−0.262	0.800	0.851
Item 35	0.735	0.400	−0.158	0.725	0.213	−0.392	0.725	0.725
Item 36	0.635	0.201	−0.069	0.448	0.281	−0.347	0.499	0.448
Item 37	0.675	0.468	0.050	0.677	0.299	−0.183	0.744	0.677
Item 38	0.718	0.299	0.123	0.621	0.435	−0.222	0.618	0.621
Item 39	0.759	0.003	0.105	0.587	0.548	−0.372	0.385	0.587
Item 40	0.549	0.200	0.109	0.353	0.352	−0.170	0.447	0.353
Item 41	0.808	0.270	0.024	0.727	0.431	−0.356	0.643	0.727
Item 42	0.321	0.324	−0.046	0.210	0.059	−0.098	0.444	0.210
Item 43	0.299	0.342	−0.148	0.228	−0.032	−0.150	0.453	0.228
Item 44	0.760	0.155	−0.005	0.602	0.420	−0.393	0.521	0.602
Item 45	0.538	0.135	0.022	0.308	0.306	−0.250	0.389	0.308
Variance	26.349	1.980	1.149	29.477	11.212	10.077	8.188	29.477
% Var	0.586	0.044	0.026	0.655	0.249	0.224	0.182	0.655

**Table 7:** Factor loading for the A-PLSI.

Factor	Indicator	Estimate	SE	Z	*p*	Stand. Estimate
Classroom interaction	Item 1	1.85	0.1053	17.53	<0.001	0.861
	Item 2	1.81	0.1096	16.50	<0.001	0.829
	Item 3	2.06	0.1168	17.63	<0.001	0.864
	Item 4	1.95	0.1098	17.73	<0.001	0.867
	Item 5	2.03	0.1138	17.82	<0.001	0.870
	Item 6	1.90	0.1073	17.72	<0.001	0.867
	Item 7	1.97	0.1257	15.63	<0.001	0.800
	Item 8	1.95	0.1165	16.75	<0.001	0.837
	Item 9	2.05	0.1264	16.22	<0.001	0.819
	Item 10	1.67	0.0976	17.12	<0.001	0.849
	Item 11	1.56	0.0973	15.99	<0.001	0.812
	Item 12	1.85	0.1096	16.86	<0.001	0.840
	Item 13	1.95	0.1281	15.21	<0.001	0.785
	Item 14	1.37	0.1092	12.55	<0.001	0.683
	Item 15	1.60	0.1188	13.43	<0.001	0.719
Social interaction	Item 16	1.39	0.0979	14.23	<0.001	0.749
	Item 17	1.44	0.0935	15.37	<0.001	0.790
	Item 18	1.55	0.0935	16.63	<0.001	0.833
	Item 19	1.95	0.1147	17.02	<0.001	0.845
	Item 20	1.68	0.1033	16.25	<0.001	0.821
	Item 21	1.48	0.0928	15.94	<0.001	0.810
	Item 22	1.64	0.0961	17.08	<0.001	0.847
	Item 23	1.64	0.0953	17.22	<0.001	0.852
	Item 24	1.68	0.1051	15.95	<0.001	0.810
	Item 25	1.87	0.1076	17.41	<0.001	0.857
	Item 26	1.95	0.1076	18.15	<0.001	0.880
	Item 27	1.72	0.1017	16.95	<0.001	0.843
	Item 28	1.62	0.0943	17.20	<0.001	0.851
	Item 29	1.92	0.1167	16.48	<0.001	0.828
	Item 30	1.91	0.1097	17.40	<0.001	0.857
Personal interaction	Item 31	1.63	0.1086	14.97	<0.001	0.778
	Item 32	1.85	0.0997	18.51	<0.001	0.892
	Item 33	1.82	0.1019	17.89	<0.001	0.874
	Item 34	1.90	0.1011	18.81	<0.001	0.900
	Item 35	1.80	0.1110	16.22	<0.001	0.821
	Item 36	1.48	0.1214	12.16	<0.001	0.669
	Item 37	1.64	0.1060	15.45	<0.001	0.795
	Item 38	1.74	0.1134	15.31	<0.001	0.790
	Item 39	1.58	0.1189	13.31	<0.001	0.716
	Item 40	1.34	0.1254	10.69	<0.001	0.604
	Item 41	1.86	0.1061	17.50	<0.001	0.862
	Item 42	1.07	0.1492	7.16	<0.001	0.428
	Item 43	1.13	0.1651	6.85	<0.001	0.411
	Item 44	1.66	0.1122	14.82	<0.001	0.773
	Item 45	1.23	0.1252	9.84	<0.001	0.564

**Table 8:** Item reliability statistics for the A-PLSI.

				If Item Dropped
Item	M	SD	Item–Rest Correlation	Cronbach’s α
Item 1	6.82	2.15	0.806	0.982
Item 2	6.79	2.19	0.789	0.982
Item 3	6.40	2.39	0.802	0.982
Item 4	6.54	2.25	0.812	0.982
Item 5	6.47	2.33	0.807	0.982
Item 6	6.64	2.20	0.811	0.982
Item 7	6.58	2.46	0.772	0.982
Item 8	6.63	2.34	0.796	0.982
Item 9	5.82	2.51	0.794	0.982
Item 10	7.11	1.97	0.815	0.982
Item 11	6.95	1.92	0.789	0.982
Item 12	6.68	2.20	0.801	0.982
Item 13	5.50	2.49	0.762	0.983
Item 14	7.04	2.01	0.677	0.983
Item 15	6.78	2.22	0.730	0.983
Item 16	6.76	1.86	0.748	0.983
Item 17	7.01	1.82	0.773	0.983
Item 18	7.12	1.87	0.813	0.982
Item 19	6.61	2.31	0.782	0.982
Item 20	6.92	2.05	0.813	0.982
Item 21	7.29	1.83	0.747	0.983
Item 22	7.06	1.94	0.780	0.982
Item 23	6.98	1.93	0.801	0.982
Item 24	7.09	2.07	0.758	0.983
Item 25	6.75	2.19	0.843	0.982
Item 26	6.64	2.23	0.846	0.982
Item 27	6.81	2.05	0.794	0.982
Item 28	7.16	1.91	0.783	0.982
Item 29	6.50	2.33	0.797	0.982
Item 30	6.65	2.23	0.820	0.982
Item 31	6.80	2.09	0.789	0.982
Item 32	7.20	2.07	0.823	0.982
Item 33	6.97	2.09	0.752	0.983
Item 34	6.92	2.11	0.785	0.982
Item 35	6.66	2.20	0.734	0.983
Item 36	6.49	2.21	0.647	0.983
Item 37	6.67	2.06	0.676	0.983
Item 38	6.72	2.20	0.717	0.983
Item 39	6.80	2.21	0.761	0.982
Item 40	6.67	2.22	0.570	0.983
Item 41	6.84	2.16	0.815	0.982
Item 42	5.56	2.50	0.361	0.984
Item 43	5.05	2.76	0.335	0.984
Item 44	6.78	2.16	0.765	0.982
Item 45	6.58	2.19	0.559	0.983

**Table 9:** Subscale reliability for the A-PLSI.

				If Item Dropped
Subscale	M	SD	Item–Rest Correlation	Cronbach’s α
Classroom interaction	98.8	28.0	0.889	0.878
Social interaction	103.3	25.9	0.892	0.874
Personal interaction	98.7	24.9	0.803	0.943

**Table 10:** Characteristics of PLD in preschoolers using the A-PLSI.

	Gender	N	Mean	SD	SE
Classroom interaction	Female	147	102.1	25.88	2.134
	Male	117	94.6	30.11	2.784
%ile rank CI	Female	147	71.2	27.63	2.279
	Male	117	71.5	31.23	2.887
Standard score CI	Female	147	12.4	3.09	0.255
	Male	117	12.5	3.41	0.316
Social interaction	Female	147	107.2	22.65	1.869
	Male	117	98.5	28.80	2.663
%ile rank SI	Female	147	66.5	28.50	2.351
	Male	117	65.8	35.35	3.268
Standard score SI	Female	147	12.6	7.48	0.617
	Male	117	16.1	14.91	1.378
Personal interaction	Female	147	102.3	22.70	1.872
	Male	117	94.2	26.84	2.481
%ile rank PI	Female	146	67.6	27.64	2.287
	Male	112	67.6	32.04	3.028
Standard score PI	Female	147	11.8	3.15	0.260
	Male	117	12.0	3.81	0.352
Pragmatic Language Development	Female	147	311.6	66.57	5.491
	Male	117	287.3	80.62	7.454
Standard score sum	Female	147	35.9	8.49	0.701
	Male	117	36.7	10.13	0.937
Pragmatic Language Index	Female	147	110.1	14.46	1.192
	Male	117	111.3	17.29	1.599

**Table 11:** Pragmatic language development and pragmatic language skills.

Dimension/Variable	Pragmatic Language Skills	N	Mean	SD	SE
Classroom interaction	Above average	62	109.3	11.67	1.482
	Average	91	84.0	13.80	1.447
	Below average	14	51.6	9.70	2.593
	Poor	6	37.0	9.38	3.830
	Superior	76	123.8	10.27	1.178
	Very poor	6	22.8	5.12	2.088
	Very superior	9	128.9	5.51	1.837
Social interaction	Above average	62	111.4	12.18	1.546
	Average	91	90.0	14.83	1.555
	Below average	14	64.9	9.46	2.529
	Poor	6	48.2	10.30	4.206
	Superior	76	126.7	8.40	0.963
	Very poor	6	30.8	13.76	5.618
	Very superior	9	131.3	3.57	1.190
Personal interaction	Above average	62	105.8	14.21	1.805
	Average	91	86.8	12.82	1.344
	Below average	14	62.7	16.18	4.324
	Poor	6	42.3	5.96	2.431
	Superior	76	120.3	11.65	1.336
	Very poor	6	32.7	3.93	1.606
	Very superior	9	125.9	7.39	2.463
Total pragmatic language skills	Above average	62	326.5	19.43	2.468
	Average	91	260.7	30.01	3.146
	Below average	14	179.1	21.80	5.827
	Poor	6	127.5	15.60	6.371
	Superior	76	370.8	22.23	2.550
	Very poor	6	86.3	19.23	7.851
	Very superior	9	386.1	9.29	3.098
Pragmatic Language Index	Above average	62	116.3	2.72	0.346
	Average	91	101.3	5.67	0.594
	Below average	14	83.9	3.18	0.851
	Poor	6	76.5	2.59	1.057
	Superior	76	125.7	2.77	0.317
	Very poor	6	65.7	4.89	1.994
	Very superior	9	133.6	2.19	0.729

**Table 12:** Pragmatic language development and age for all participants in the A-PLSI.

Dimension/Variable	Age in Years	N	Mean	SD	SE
Classroom interaction	4	29	71.2	30.2	5.600
	5	27	82.4	33.8	6.508
	6	60	100.8	26.7	3.452
	7	148	106.3	22.0	1.810
Social interaction	4	29	74.9	28.2	5.234
	5	27	89.6	29.6	5.694
	6	60	105.3	25.0	3.225
	7	148	110.6	19.8	1.627
Personal interaction	4	29	79.6	30.0	5.570
	5	27	88.2	31.6	6.090
	6	60	102.4	23.5	3.037
	7	148	102.9	20.6	1.689
Total pragmatic language skills	4	29	225.7	84.8	15.745
	5	27	260.1	92.8	17.861
	6	60	308.4	72.3	9.330
	7	148	319.9	54.7	4.499
Pragmatic Language Index	4	29	95.8	18.0	3.345
	5	27	101.4	19.5	3.744
	6	60	113.3	15.4	1.986
	7	148	114.2	12.1	0.993

## Data Availability

The data presented in this study are available on request from the corresponding authors. The data are not publicly available due to ethical restrictions.

## References

[1] Qasem F., Alduais A., Alfadda H., Alfadda N., Al Amri L. (2022). A Study on the Relationship between Pragmatic Language Development and Socioeconomic Status in Arab Preschoolers with and without Pragmatic Language Impairment. Sustainability.

[2] Gleason J.B., Ratner N.B. (2016). The Development of Language.

[3] Kuder S.J. (2018). Teaching Students with Language and Communication Disabilities.

[4] Parker F., Riley K. (2010). Instructor’s Manual Linguistics for Non-Linguistics A Primer with Exercises.

[5] Martin I., McDonald S. (2003). Weak Coherence, No Theory of Mind, or Executive Dysfunction? Solving the Puzzle of Pragmatic Language Disorders. Brain Lang..

[6] O’Neill D.K. (2007). The Language Use Inventory for Young Children: A Parent-Report Measure of Pragmatic Language Development for 18- to 47-Month-Old Children. J. Speech Lang. Hear. Res..

[7] Ciccia A.H. (2018). Pragmatic Communication. Encycl. Clin. Neuropsychol..

[8] Turkstra L.S., Clark A., Burgess S., Hengst J.A., Jeffrey C., Paul D., Turkstra L.S., Clark A., Burgess S., Hengst J.A. (2017). Pragmatic Communication Abilities in Children and Adults: Implications for Rehabilitation Professionals. Disabil. Rehabil..

[9] Ervin-Tripp S., Guo J., Lampert M. (1990). Politeness and Persuasion in Childen’s Control Acts. J. Pragmat..

[10] Bierman K.L. (2004). Peer Rejection: Developmental Processes and Intervention Strategies.

[11] Diken Ö. (2014). Pragmatic Language Skills of Children with Developmental Disabilities: A Descriptive and Relational Study in Turkey. Eurasian J. Educ. Res..

[12] Park C.J., Yelland G.W., Taffe J.R., Gray K.M. (2012). Brief Report: The Relationship Between Language Skills, Adaptive Behavior, and Emotional and Behavior Problems in Pre-Schoolers with Autism. J. Autism Dev. Disord..

[13] Walker V.L., Snell M.E. (2013). Effects of Augmentative and Alternative Communication on Challenging Behavior: A Meta-Analysis. Augment. Altern. Commun..

[14] Chiang H. (2008). Expressive Communication of Children with Autism: The Use of Challenging Behaviour. J. Intellect. Disabil. Res..

[15] Cocquyt M., Mommaerts M.Y., Dewart H., Zink I. (2015). Measuring Pragmatic Skills: Early Detection of Infants at Risk for Communication Problems. Int. J. Lang. Commun. Disord..

[16] Sante D., Sylvestre A., Bouchard C., Leblond J. (2019). The Pragmatic Language Skills of Severely Neglected 42-Month-Old Children: Results of the ELLAN Study. Child Maltreat..

[17] Hyter Y.D. (2021). Childhood Maltreatment Consequences on Social Pragmatic Communication: A Systematic Review of the Literature. Perspect. ASHA Spec. Interest Groups.

[18] Sylvestre A., Bussieres E.-L., Bouchard C. (2015). Language Problems Among Abused and Neglected Children: A Meta-Analytic Review. Child Maltreat..

[19] Sylvestre A., Mérette C. (2010). Child Abuse & Neglect Language Delay in Severely Neglected Children: A Cumulative or Specific Effect of Risk Factors?. Child Abuse Negl..

[20] Gilliam J.E., Miller L. (2006). PLSI: Pragmatic Language Skills Inventory.

[21] Alev G., Diken I.H., Ardiç A., Diken O., Şekercioǧlu G., Gilliam J. (2014). Adaptation and Examining Psychometrical Properties of Pragmatic Language Skills Inventory (PLSI) in Turkey. Elem. Educ. Online.

[22] Diken Ö. (2019). Describing and Comparing Pragmatic Language Skills of Turkish Students with Typical Development and Inclusive Education Students with Mild Intellectual Disability. Int. J. Progress Educ..

[23] Alduais A., Wendt A.N. The Development of Infant Language in the First 12 to 42 Months of Life: A Thematic Review of Protective and Risk Factors. Proceedings of the International Psychological Applications Conference and Trends.

[24] Dicataldo R., Roch M. (2021). Direct and Indirect Pathways of Variation in Length of Exposure to the Majority Language, Cognitive and Language Skills in Preschoolers’ Listening Narrative Comprehension. Children.

[25] Joensuu E., Munck P., Setänen S., Lipsanen J., Huhtala M., Lapinleimu H., Stolt S.K.J. (2021). Associations between Language at 2 Years and Literacy Skills at 7 Years in Preterm Children Born at Very Early Gestational Age and/or with Very Low Birth Weight. Children.

[26] Ching T.Y.C., Cupples L., Leigh G., Hou S., Wong A. (2021). Predicting Quality of Life and Behavior and Emotion from Functional Auditory and Pragmatic Language Abilities in 9-Year-Old Deaf and Hard-of-Hearing Children. J. Clin. Med..

[27] Dicataldo R., Florit E., Roch M. (2020). Fostering Broad Oral Language Skills in Preschoolers from Low SES Background. Int. J. Environ. Res. Public Health.

[28] Dicataldo R., Roch M. (2020). Are the Effects of Variation in Quantity of Daily Bilingual Exposure and Socioeconomic Status on Language and Cognitive Abilities Independent in Preschool Children?. Int. J. Environ. Res. Public Health.

[29] Van den Bedem N.P., Dockrell J.E., van Alphen P.M., Rieffe C. (2020). Emotional Competence Mediates the Relationship between Communication Problems and Reactive Externalizing Problems in Children with and without Developmental Language Disorder: A Longitudinal Study. Int. J. Environ. Res. Public Health.

[30] Andrés-Roqueta C., Garcia-Molina I., Flores-Buils R. (2021). Association between CCC-2 and Structural Language, Pragmatics, Social Cognition, and Executive Functions in Children with Developmental Language Disorder. Children.

[31] Alduais A.M., Shoeib R.M., Al Hammadi F.S., Al Malki K.H., Alenezi F.H. (2012). Measuring Pragmatic Language in Children with Developmental Dysphasia: Comparing Results of Arabic Versions of TOPL-2 and CELF-4 (PP and ORS Subtests). Int. J. Linguist..

[32] Khodeir M.S., Hegazi M.A., Saleh M.M. (2018). Development and Standardization of a Test for Pragmatic Language Skills in Egyptian Arabic: The Egyptian Arabic Pragmatic Language Test (EAPLT). Folia Phoniatr. Logop..

[33] Searle J. (1969). Speech Acts: An Essay in the Philosophy of Language.

[34] Bates E. (1976). Language and Context: The Acquisition of Pragmatics.

[35] Austin J.L., Urmson J.O., Sbisà M. (1962). How to Do to Things with Words.

[36] Alduais A., Shoeib R., Al-Hammadi F., Al-Malki K. (2012). Testing the Usability of an Arabic Version of TOPL-2 in Measuring Pragmatic Language Impairment in Children and Adolescents with Developmental Dysphasia. Int. J. Linguist..

[37] Alduais A. (2012). Investigating the Relationship between Pragmatic Language Development and Early Childhood Education: A Correlational Study on a Sample of Saudi Female Preschoolers and Nonpreschoolers. IOSR J. Humanit. Soc. Sci..

[38] Alduais A.M.S. (2012). Use of an Arabic-Language Version of TOPL-2 to Identify Typical and Atypical Manifestations of Pragmatic Language Impairment in Individuals with Developmental Dysphasia. IOSR J. Humanit. Soc. Sci..

[39] Alduais A.M.S. (2013). Identifying Typical and Atypical Manifestations of Pragmatic Language Impairment in Arabic: A Multi-Method Study of Individuals with Developmental Dysphasia. Master’s Thesis.

[40] Alkadhi A. (2015). Assessing Early Sociocognitive and Language Skills in Young Saudi Children. Ph.D. Thesis.

[41] Phelps-Terasaki D., Phelps-Gunn T. (2007). TOPL-2: Test of Pragmatic Language.

[42] O’Neill D. (2009). Language Use Inventory LUI.

[43] American Speech-Language-Hearing Association Social Communication Disorder. https://www.asha.org/practice-portal/clinical-topics/social-communication-disorder/#collapse_1.

